# Supplementary material for: Geochemical and metagenomics study of a metal-rich, green-turquoise-coloured stream in the southern Swiss Alps
Source: PLoS One. 2021 Mar 30;16(3):e0248877. doi: 10.1371/journal.pone.0248877 (PMC8009434; doi:10.1371/journal.pone.0248877)
Supplement: Attachment — Submitted filename: Response_to_Reviewers.pdf [file pone.0248877.s007.pdf]

SUPSI

Dr. Antoine Buetti-Dinh,
e-mail: antoine.buetti@supsi.ch

Editor,
PlosOne

Bellinzona, January 12, 2021

Dear Editor,

Please find our revised manuscript that includes modifications according the reviewing report PONE-D-20-23974, together with our replies (pasted below) addressing each issue raised by the Reviewer.

The revised manuscript changes mentioned in this reply letter are highlighted with colours and referred to line numbers or sections of the new manuscript version.

We have made considerable efforts to reply to the Reviewer's comments. Following these modifications, and given that the Reviewer was overall positive with the manuscript, we hope that the manuscript can be considered in its present form, and would like to thank you and the Reviewer for the careful attention in reading and evaluating our manuscript.

On behalf of all authors,
Antoine Buetti-Dinh, SUPSI, Bellinzona, Switzerland

Journal Requirements

Journal Requirement 1: Please ensure that your manuscript meets PLOS ONE's style requirements, including those for file naming.

Reply: Style requirements follow Plos One guidelines as indicated in the current web page links.

Journal Requirement 2: PLOS requires an ORCID iD for the corresponding author in Editorial Manager on papers submitted after December 6th, 2016.

Reply: The corresponding author's ORCID (0000-0002-6469-0296) is linked to his Plos One profile in the Editorial Manager as requested.

Journal Requirement 3: Please upload a copy of Supporting Information S1 File, S2 File and S3 File which you refer to in your text on page 8.

Reply: The Supporting Information files are available at the links indicated on page 8 of the main text. Since these include an interactive diagram and metagenome sequences, we opted not to upload the corresponding files to the Editorial Manager (format not supported). The only piece that could be provided also in the Editorial Manager is S1 File, a technical report in format ".doc". An additional table requested by this revision round (S4 File) has also been uploaded in the Editorial Manager.

Additional Editor Comments

Additional Editor Comments 1: General. The reviewer has made very good comments. Please carefully check the phrasing to avoid over-interpretation. As the absolute must-do in your case is the deposition of the data in a public library. Since that may some time, I would decide on major revision (to give you time), although from the content of the expert reviewer's comments it would qualify for minor revision.

Reply: We thank the Reviewer for his very helpful and encouraging comment. Raw data have been deposited as a SRA/BioProject accessible through the Accession Number PRJNA689378.

Reviewers' comments

Comment 1: This manuscript presents a metagenome study of metal-rich aquatic envi-

ronment in the Swiss alps. What a terrific field site! This is a great focused study using analytical profiling of the water as well as metagenome study of the microbial mats to understand community makeup.

Reply: We thank Prof. Stajich for the positive comment, we also think it is a quite special study site, and plan further studies by monitoring seasonal changes over the coming years.

Abstract

Comment 1: "genes that have been selected to allow microbial adaptation" → Clarify why you are surprised to find EPS genes, aren't these known to be encoded in Cyanobacteria genomes?

Reply: It is right, EPS genes are encoded in Cyanobacteria genomes. We were not surprised to find EPS genes, we just described the genetic elements found in the metagenome with know functions with respect to chemical conditions found on site. As discussed in section "Potential and limitations", we acknowledge that this fact does not necessarily provide evidence for actual activity of the genes found in the metagenome. We reformulated the portion of the abstract which leads the reader to think that we were surprised to find EPS genes, as well as the last sentence of the "Introduction" section.

Comment 2: Some of the language is a little imprecise and could be helpful to improve. Line 2: "are composed of different biofilms of microorganisms" - could you be more clear is there one biofilm comprised of many organisms, or are there layers of films each heterogeneous compositions of microbes?

Reply: Thanks for the comment. We indeed wanted to point out that microbial mats are layers of films of heterogeneous compositions of microbes, paraphrasing the article cited as reference (ref.4) which says "Microbial mats are horizontally stratified microbial communities". We rephrased the sentence accordingly (line 2).

Methods

Comment 3: Please provide version numbers for the tools used (MetaMaps, Krona Tools) some information about what version the miniSeq+H database was searched to assign taxonomic names?

Reply: Thanks for noticing this missing information that went mistakenly unnoticed by us (lines 95-96).

Comment 4: Not clear why pacbio-raw was used when running ONT reads, isn't -nanopore-raw is an option?

Reply: We thank the Reviewer for noticing this incongruence. This option (line 100) was simply chosen by mistake as suggested by the default command line for assembling metagenomes with the canu tool, and interpreted to be part of the adjustments needed to the canu suite for handling metagenomes. We have repeated the whole procedure using the –nanopore–raw option, and validated all the claims in the results section. The assembled reads differed, and the gene annotation also had some differences whose implications did not change the conclusions of the manuscript. Of course, each such difference was integrated in the "Results and discussion" session (in purple color). In addition, the annotation file provided as supplementary information has been updated accordingly.

Comment 5: The canu option listed 5m - so is that reasonable genome size for a metagenome? I suppose it is just an estimate to get depth of coverage correct for how it runs- but you might evaluate after the assembly whether contigs have different depth of coverage values indicating organisms in different abundances in the sample.

Reply: In the canu tool, this option refers to the average genome length in the sample, and not to the full length of the assembled metagenome. This option, along with the rest of the parameters used, were suggested as default for metagenomes in the current canu guidelines. We have tested the procedure with an average genome length of 50m, but the procedure failed after several weeks and the generation of >800GB of metadata that exceeded our machine capacity. This, according to the developer, should not affect the outcome of the procedure, as stated in `https://github.com/marbl/canu/issues/634` ("assembly with a large genome size [is] comparable to one with a small one but using larger compute nodes"). Further, we also corrected for coverage of the different contigs by the applying the binning procedure as suggested (see "Comment 7" and "Comment 11"). The coverage between the different bins varies roughly between 10 and 200 and is reported along with other parameters of the binning procedure in the newly added supplementary table (S4 File). The outcome does not appear to influence substantially the conclusions, as discussed below.

Comment 6: What is "the blast database" indicated on line 101?

Reply: This referred to the "NCBI BLAST's nt database" (v2.10.0+). We have specified this accordingly in the new manuscript version (line 102).

Comment 7: I'm surprised no metagenome binning applied to better adjust for individual species genomes?

Reply: Metagenome binning has been applied as suggested. The results of CONCOCT (v1.1.0) suite assigned the assembled contigs to 36 bins corresponding to the identified Metagenome-Assembled Genomes (MAGs) discussed below in "Comment 11", while the MetaBAT2 (v2.15) suite in a single bin. Therefore, the MetaBAT2 results have not been further processed. The results of the binning procedure has been reported in the newly added supplementary table (S4 File).

Comment 8: line 230: "that take profit of long reads" → this phrasing could be "profit from" or "take advantage of".

Reply: We changed this accordingly (line 242).

Comment 9: Line 233: "potential gene program", the term "gene program" is a little confusing – but just may depend on how you want to word it.

Reply: We rephrased the sentence and used the term "genetic composition" instead of "potential gene program" (line 245).

Comment 10: It seems helpful to spend a little more time contrasting the levels of chemicals found with those in other aquatic environments to clarify how extreme this makeup is? The results/discussion do not cover extensively an interpretation of the quantitative values found - are they at extremes of most life? are they beyond what is found in most streams?

Reply: We added a paragraph in the "Hydrochemical and geochemical analyses" section ("Results and discussion") that summarizes the findings by comparing the metal concentrations found on the study site with the concentration ranges found in typical Alpine environments. In short, they are not at the extremes of life, but beyond to the usual ranges of copper, cadmium and zinc found in the Alps.

Comment 11: The authors' argument that the microbes in the community have adapted by selection to the environment are attractive but lack much statistical rigor. Just counting up genes without contrasting to an alternative model isn't sufficient. For example - if you focused on one of the most well assembled microbes (again a benefit to binning the contigs to species so you can assess the overall gene content of one of the Metagenome-Assembled Genome (MAG) - you could contrast the copy number of metal resistance implicated genes or transporter genes with the gene set found in a sister lineage which was not from non-

extreme conditions.
A. if you do this, then some assessment of completeness of the genomes - eg BUSCO or CheckM scores.
B. annotation of each of these individually may provide slight better results if gene predictors were able to run and train on each genome set individually. To that end I am not sure if Prokka would perform better on the annotation if the data were binned and run each one at a time.

Reply: We agree that the reader might be misled in thinking that we present arguments for individual microbes' adaptation to the study site's environment. We corrected this misunderstanding in the abstract sentence "genes that have been selected to allow microbial adaptation in such exceptional environment" also according to "Comment 1". This should make clear that the microbial community is adapted to those particular environmental conditions, rather than that the microbes in the community have adapted by selection to the environment, a statement that cannot be made without comparing our single dataset to other time points/study sites. Metagenome binning was carried out as requested, using the CONCOCT (v1.1.0) suite that generated 36 bins corresponding to the identified MAGs. These were evaluated with CheckM for completion and contamination reported in the newly added supplementary table (S4 File) and annotated MAGs (S3 File) along with a pragraph in the "Results" section ("MinION metagenomics sequencing analysis") (line 145). Unfortunately, CheckM could only return estimates down to at most the Phylum of the detected organisms, not allowing us to compare the number of gene copies to sister lineages from non-extreme conditions. Indeed, MAGs annotation with Prokka allowed the identificaton of few other subunits of the genes previously described in the main text. Each such difference was integrated in the "Results and discussion" section (in brown color), "Overall bacterial diversity" (lined 183-184).

Comment 12: Were there any evidence of archaea or non-bacteria in your metagenomes?

Reply: Yes, as reported in the interactive Krona diagram available as supplementary information, among all organisms identified, 0.3% were Archaea, 2% were Eukaryota (of which *Homo sapiens* constituted 64%), and another 2% were viruses.

Comment 13: Just to comment on this – I believe there may be signal towards understanding if adaptation has occurred but that would be better addressed with something quantifiable – eg accelerated rates of molecular evolution; expansions of copy number of gene families that underlie EPS or metal tolerance.

Reply: Determining rates of evolution would require to study genes, gene regulatory mechanisms and enzymes involved in the metabolism of the chemical components found on the study site. In particular, this would require the comparison of such biological elements with respect to the same species living elsewhere, which would serve as a reference from which one could argue if mechanisms of adaptation or accelerated evolution have taken place in the study site

(such a purifying or positive selection observed at the level of the molecular components in the genomes of the organisms living there). In a simpler experimental setup, the copy number of genes involved in the metabolism of the chemical components found in a given organism on the study site could be used as an argument for its adaptation to the actual conditions. In an even simpler experimental setup, the presence/absence of genes over the whole genomes found on the site, could be used as an indication that organisms have been selected to the particular conditions. For example finding genes for life under high pressure or high osmotic conditions might be correlated to particular environmental conditions that selects them. The latter, simplest scenario, is in line with our present study. As mentioned earlier, we did not want to give the idea that we have observed adaptation of individual microbes to that environment, we only present a descriptive study based on chemical and microbiological compositions of a biomat, which altogether as a biological entity, is adapted to that particular environment (perhaps not very surprisingly, once it is known to be there).

Comment 14: Data availability – The metagenome raw data and annotated assembly much be deposited in the INSDC public sequence archive (genBank, EMBL, DDBJ). Supl file 3 is not a substitute for depositing in a sequence archive. Likewise the raw fast5 data from ONT need to be deposited into SRA and a BioProject and SRA project ID assigned to the unassembled dataset.

Reply: Raw data have been deposited as a SRA/BioProject accessible through the Accession Number PRJNA689378.
